# Nontypical presentation of a common disease: a case report

**DOI:** 10.1186/s13256-021-03219-0

**Published:** 2022-01-13

**Authors:** Walaa Alshammasi, Abeer Bargawi, Aljuhara Abdulrahman, Mariam Alhaji, Fakherah AL Qahtani, Ali Aldajani

**Affiliations:** 1 Maternal and Children Hospital in Dammam, Dammam, Saudi Arabia; 2 Sick Kids Hospital, Toronto, ON Canada

**Keywords:** Kawasaki disease, AKI, Renal involvement, Vasculitis

## Abstract

**Background:**

Kawasaki disease is an idiopathic medium-sized vasculitis that occurs primarily in infants and children younger than 5 years of age. Atypical Kawasaki disease applies to patients who do not fulfill the complete criteria of fever of 5 days or more with at least four of five features: bilateral conjunctival injection, changes in the lips and oral cavity, cervical lymphadenopathy, extremity changes, and polymorphous rash. *Acute kidney injury* is defined as a sudden decline in kidney function within hours, including structural injuries and loss of function. Acute kidney injury is extremely common in hospitalized pediatric patients. However, it is rarely documented in Kawasaki disease. Acute kidney injury is underestimated in Kawasaki disease due to the lack of a clear definition of age-specific normal serum creatinine levels and routine renal functions. This report describes a case who presented with clinical features suggestive of atypical Kawasaki disease and developed acute kidney injury.

**Case presentation:**

A 2-year-old Saudi girl had a history of high-grade fever for 5 days, moderate dehydration, dry cracked lips, poor appetite, and generalized erythematous rash; therefore, she was diagnosed to have incomplete Kawasaki disease. Laboratory investigations revealed normochromic normocytic anemia, leukocytosis, thrombocytosis, high inflammatory markers, and acute kidney injury stage III. An echocardiogram showed a 4-mm dilatation on the left main coronary artery and a 3-mm dilatation on the right. A renal biopsy was not performed to identify the cause of the injury as it showed improvements after the start of the specific therapy for Kawasaki disease; intravenous immune globulin at a dose of 2 g/kg, aspirin at a high dosage of 80 mg/kg/day, and prednisolone at 2 mg/kg. In addition to the acute kidney injury management, normal saline boluses were followed by furosemide at a 2 mg/kg dose. Her urine output increased, and her renal functions normalized. She was discharged in good condition after 10 days.

**Conclusions:**

It is valuable to check renal function tests in a confirmed case of Kawasaki disease to reduce the negative consequences of late acute kidney injury discovery. Early detection and intervention make a substantial difference in acute kidney injury management**.**

## Background

Tomisaku Kawasaki (1967) was the first to introduce Kawasaki disease (KD). Since then, it has been recognized as a critical childhood disease worldwide, as an idiopathic acute systemic inflammatory disease. KD involves a medium-sized vasculitis that occurs mainly in infants and children younger than 5 years of age [1–3, 22].

The identification of KD is established based on at least four criteria: polymorphous skin rash, changes such as swelling of the hands and feet (erythema, edema, or peeling), bilateral nonexudative bulbar conjunctival injection, changes in the lips and oral cavity, and nonpurulent cervical lymphadenopathy of 1.5 cm, in addition to fever for at least 5 days. Only a small subgroup of patients does not fulfill the full criteria, and are diagnosed with atypical KD [1–4, 22].

The prevalence of the disease is highest in Japan, Korea, and Taiwan, followed by the rest of the Asian countries [2, 4, 5, 16–18, 22].

Sterile pyuria and non-nephrotic range proteinuria are the most common kidney complications, and other kidney complications are uncommon [1, 8–11]. Although acute kidney injury (AKI) is a common complication in hospitalized children, it has rarely been reported in patients with KD.

In reference to Kidney Disease: Improving Global Outcomes (KDIGO), AKI is determined as an increase in serum creatinine (SCr) levels by 0.3 mg/dL within 8 hours, or an increase in SCr levels by 1.5 times from baseline, which is known or presumed to have occurred within the previous 7 days, or with a urine volume of less than 0.5 mL/kg/hour for 6 hours [13].

The presentation of AKI in KD has not been documented in the Kingdom of Saudi Arabia. Here, we report a novel case of a Saudi child with clinical features suggestive of KD who developed AKI. To the best of our knowledge, no similar case has been reported in an Arab child. We believe this case contributes to the literature; to date, it is unknown whether AKI in KD varies between ethnicities, age groups, and geographical areas. Furthermore, whether it is a rare complication in KD or underestimated due to lack of routine renal function tests in KD patients, limited published data, or the lack of a clear definition of age-specific average SCr values [3, 5, 6, 17, 18].

## Case presentation

A previously healthy 2-year-old Saudi girl was admitted to the hospital due to 5-day history of high fever (body temperature 38.5–39.5 °C, bilateral leg pain that prevented her from walking, fatigue, generalized erythematous rash, cracked lips, and reduced appetite.

Her nutritional history before this episode was appropriate for her age, and she began to share a family diet at the age of 1 year. Her calories intake were around 90–100 kcal/kg/day. The fluid intake range was (1.5–2 L)/day.

**Fig. 1:**
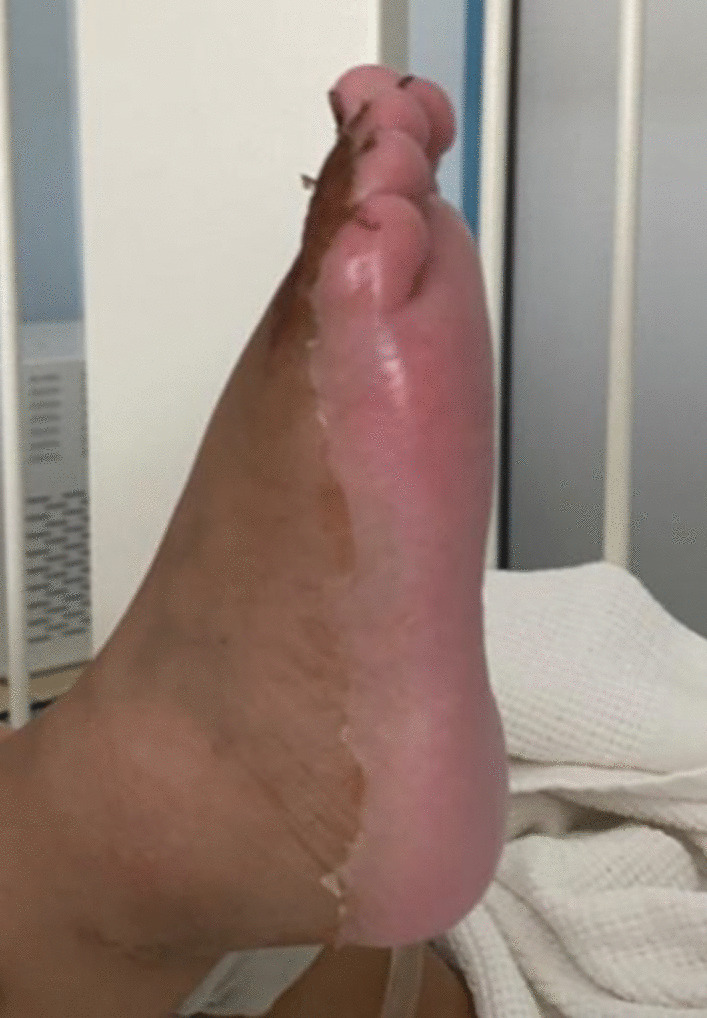
Desquamation of the skin on the right sole

**Fig. 2:**
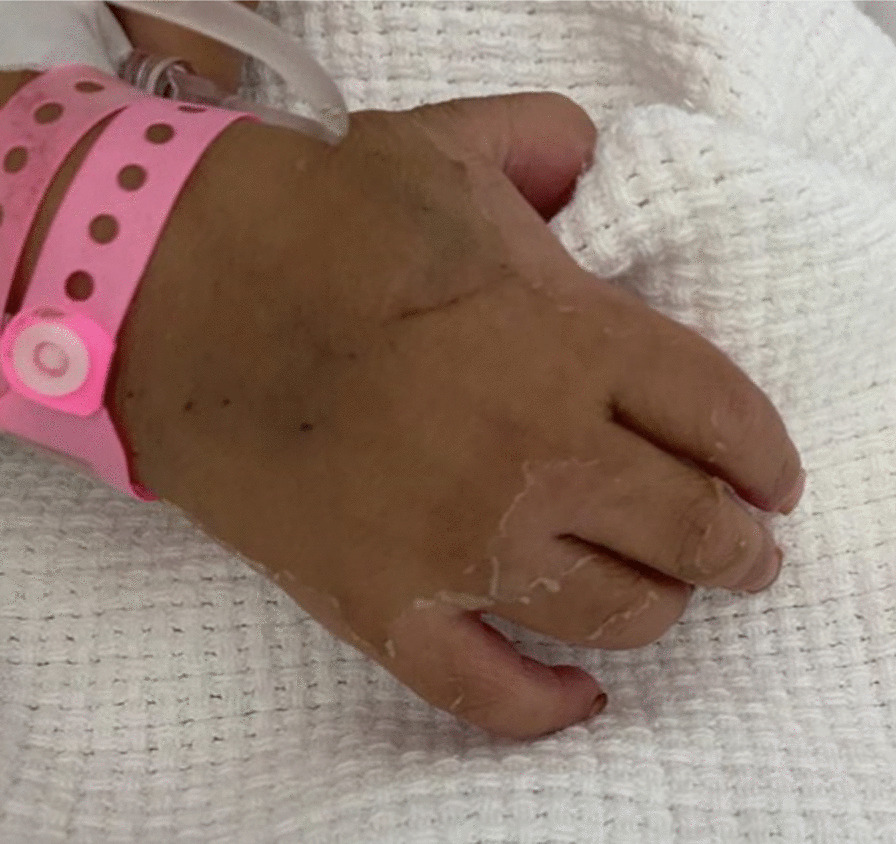
Desquamation of the skin on the right palm and extending to the dorsal hand involving the fingers

Furthermore, she developed skin desquamation on the seventh day of admission on her palms, soles, and genitals (Figs. 1, 2). There was no history of conjunctivitis, neck swelling, upper respiratory symptoms, vomiting, diarrhea, headache, or neurological symptoms. There was no history of coronavirus disease (COVID-19) within the 4 weeks prior to her presentation. She has no previous medical or surgical history, and was not on regular medication.

Developmentally, she is up to her age. She kicks a ball, draws a line, says sentences of two words, and gets excited with children. Concerning family history, parents and the younger sibling were healthy, without a similar presentation, no history of chronic illnesses, no history of vasculitis or kidney disease.

**Fig. 3:**
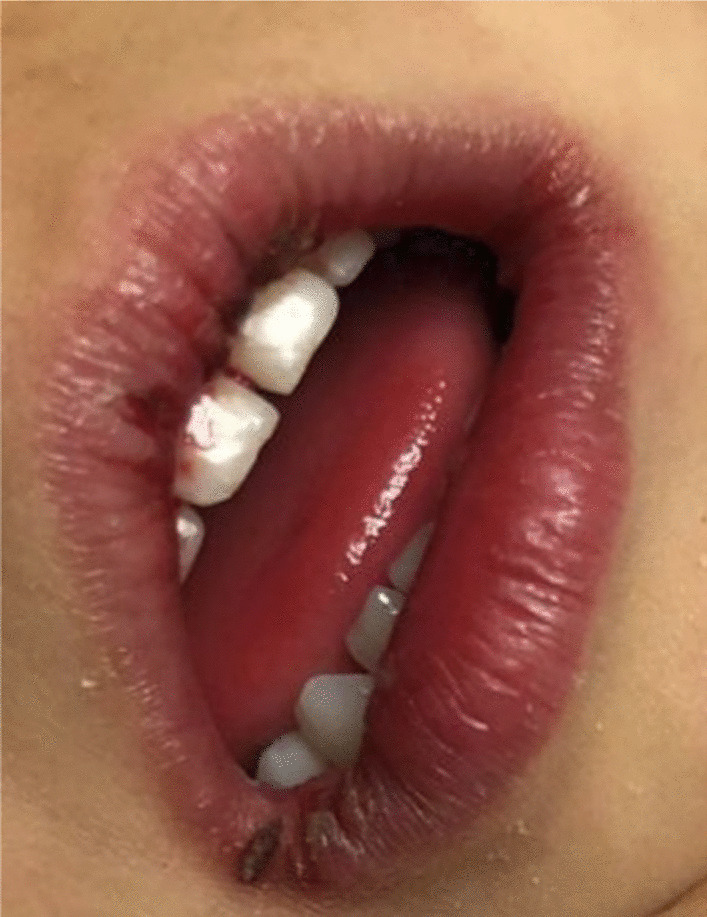
Dry cracked lips

The physical examination revealed that the child was irritable, moderately dehydrated (10%), and had dry lips (Fig. 3). She looked well-nourished with an average body build.

Vital signs showed a temperature of 38.5 °C, blood pressure of 103/71 mmHg, pulse rate of 104 beats per minute, admission weight of 16 kg (95th percentile), and height of 94 cm (85th percentile). The examination of the skin revealed a generalized maculopapular rash. Both hands and feet were swollen, and she had thickened skin on both palms and soles covered by henna. There was no cervical adenopathy, lung congestion, or abnormal heart sounds. The abdomen was soft, had no hepatosplenomegaly, and the rest of the examination results were unremarkable.

Clinically, she met the criteria for incomplete KD.

Initial laboratory investigation showed normochromic normocytic anemia, normal counts of platelets, and white blood cells on the higher side (Table 1). Renal function was impaired, with high levels of urea and SCr (Table 2). The electrolyte levels were as follows: sodium (134 mmol/L), potassium (4.2 mmol/L), calcium (2 mmol/L), phosphate (1.5 mmol/L), and magnesium (0.9 mmol/L). The hepatic profile showed normal levels of alanine transaminase (27 U/L), aspartate transaminase (34 U/L), albumin (range: 24–26 g/L), total bilirubin (1 mg/dL), and direct bilirubin (0.3 mg/dL), and a normal coagulation profile.

**Table 1:** Hematology and inflammatory markers lab results during admission

	HGB g/dL (10.6–14.5)	PLT 10^3^/uL (150–440)	WBC 10^9^/L (6.0–16.0)	ESR mm/hour (3–13)	CRP mg/L (0.0–5.0)
22/06/2020	9	258	11.4	119	85.6
24/06/2020	7.4	577	11.2	150	54.7
26/06/2020	7.6	1154	11.6	118	18.1
28/06/2020	7.7	1325	15.8	128	15
30/06/2020	8.7	1542	14.3	85	4.20

*HGB* hemoglobin, *PLT* platelet, *WBC* white blood cell, *ESR* erythrocyte sedimentation rate, *CRP* C-reactive protein

**Table 2:** Renal Function Tests progression during admission

Day of admission	Creatinine μmol/L (53.1–97.3)	Urea mmol/L (3.6–17.9)
First	349	35
Second	453	37.2
Third	439	33.6
Fourth	225	20
Fifth	116	11.1
Sixth	64	4.9
Seventh	62	3.9
Eighth	60	4
Ninth	58	5.2
Tenth	52	4.4

Levels of inflammatory markers were high (Table 1). Additionally, ferritin and D-dimer levels were high (381 µg/L and 3 µg/mL, respectively). Fibrinogen, lactate dehydrogenase (LDH), procalcitonin, and troponin levels were normal.

Blood culture, throat swab, COVID-19 test swab, and serology [severe acute respiratory syndrome coronavirus 2 (SARS-CoV-2) immunoglobulin G (IgG)] showed negative results.

Urine analysis and urine culture revealed sterile pyuria and hematuria (Table 3).

**Table 3:** Urine analysis results

Urine analysis
WBC: > 100/HPF
RBC: 6–10/HPF
Protein: negative
Hemoglobin: 3+
Ketone/glucose: negative

*WBC* white blood cell, *RBC* red blood cell, *HPF* high-power field

Reference ranges: WBC (negative), RBC (negative–5)/HPF, protein (negative–trace), hemoglobin (negative), ketones (negative), glucose (negative)

**Fig 4:**
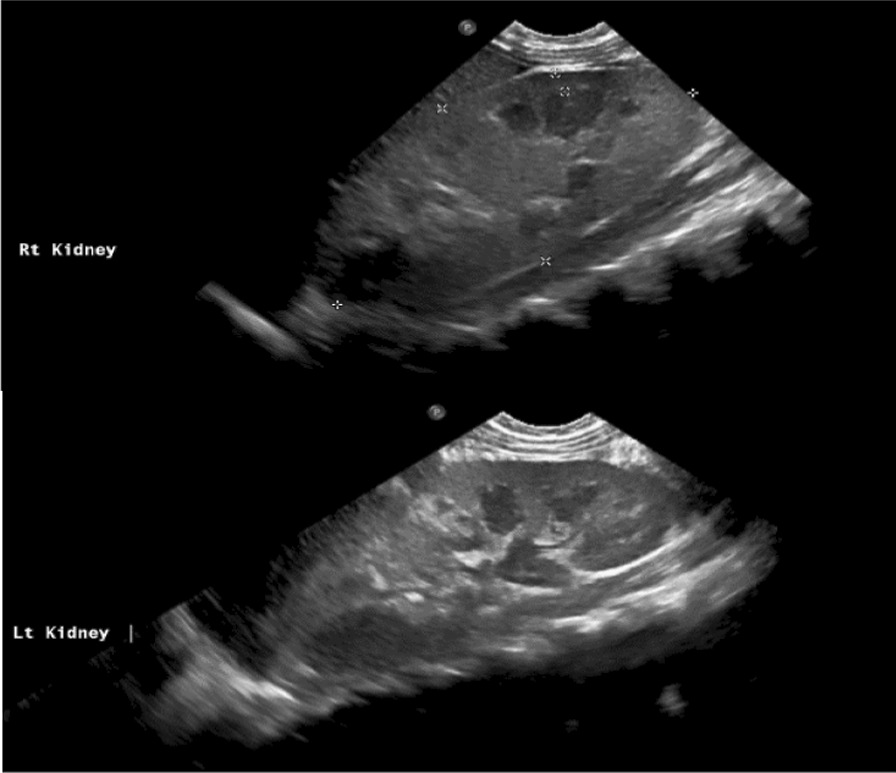
Enlarged kidneys with poor corticomedullary differentiation, increased echogenicity, and no cysts or stones

Abdominal ultrasound showed enlarged kidneys with poor corticomedullary differentiation, increased echogenicity, and no cysts or stones (Fig. 4).

Chest radiography revealed cardiomegaly, and electrocardiography showed a prolonged PR interval (measured from the beginning of the P wave to the beginning of the QRS complex); a two-dimensional echocardiogram initially showed normal results. However, the platelet count increased over the days of hospitalization. This was associated with leukocytosis, increased inflammatory markers (Table 1), and excessive peeling that developed on both palms and soles. Therefore, an echocardiogram was repeated that showed a 4-mm dilatation on the left main coronary artery and a 3-mm dilatation on the right.

KD was diagnosed based on echocardiogram findings, in addition to meeting three of the six criteria. There were no funding or cultural challenges regarding this diagnosis and she received intravenous immunoglobulin (2 g/kg) and a high dosage of aspirin (80 mg/kg/day) and prednisolone (2 mg/kg), and she was discharged after 2 weeks.

On the third day of admission, the patient had an increase in blood pressure to 129–134/55–60 mmHg (> 95th + 12 mmHg percentile), an increase in weight by 0.5 kg, an increase in SCr levels [glomerular filtration rate (GFR): 7.5 mL/minute/1.73 m^2^] (Table 2), which put her in stage III AKI based on KDIGO guidelines, and a decrease in sodium levels to 127 mmol/L. A urinary catheter was then inserted with strict input–output charting.

She had oliguria, in which her urine output was less than 1 mL/kg/hour, with a positive balance of 400–500 mL. Broad-spectrum antibiotics and normal saline boluses were administered, followed by furosemide challenge at a dose of 2 mg/kg. Subsequently, her urine output increased to 2 mL/kg/hour and reached 4 mL/kg/hour the next day. Her GFR recovered (Table 2), blood pressure normalized, and repeat serum sodium level was 133 mmol/L.

After 10 days of admission, the patient was discharged in good condition with a discharge weight of 15.9 kg and normal blood pressure. She attended regular follow-up in the nephrology clinic, where her blood pressure readings were normal, and her last documented creatinine was 45 μmol/L.

## Discussion

In our case, the onset of fever, followed by cracked lips, generalized rash, swollen hands and feet, and extensive peeling in the erythematous perineal area occurs in the acute phase of KD, which typically lasts 10–14 days.

Diffuse erythema in the genital area that peels during the acute phase is seen in up to half of patients. The average platelet count and absence of coronary artery dilatation initially indicate the acute clinical phase.

The subacute phase began when the fever was resolved, the skin was desquamated in the hands and feet, and the increase in platelets over time required repeated echocardiography, which showed the characteristic condition at this stage: coronary artery dilatation [1, 20, 22].

Since KD is a systemic vasculitis, there can be several organs involved. Coronary lesions are the most severe and common complications in KD, which occur in 25% of untreated children [5–7, 22]. During the COVID-19 pandemic, the suspicion of a multisystemic inflammatory syndrome in children (MIS-C) is logical after a history of fever lasting more than 24 hours in individuals aged < 21 years, with evidence of inflammation (elevated levels of erythrocyte sedimentation rate, C-reactive protein, ferritin, and D-dimer); severe illness requiring hospitalization with multiple systems involving more than two systems, including echocardiogram findings; AKI; and dermatologic findings (rash, dry lips with crack), with fulfillment of partial criteria for atypical KD.

MIS-C criteria must include positive results for current or recent SARS-CoV-2 infection detected on polymerase chain reaction, serology, or antigen testing or COVID-19 exposure within 4 weeks prior, which were all negative in the present case. Furthermore, normal fibrinogen, procalcitonin, and LDH do not support the diagnosis [19].

As for renal involvement in KD, sterile pyuria is the most common urinary presentation, followed by various other manifestations, such as proteinuria and hematuria [1, 8–11]. It is unsurprising that the patient, in this case, had sterile pyuria and microscopic hematuria (Table 3).

AKI is rarely associated with KD. Interestingly, Chuang *et al*. investigated the clinical features and data of 336 Taiwanese patients with KD, including their serum creatinine (SCr) levels, and reported that 28% of them developed AKI. They linked the risk to two factors: the young age, which applies to our case, and the high alanine transaminase level, which does not apply to our case, as she had normal levels [12].

Mousa *et al*. [2] were the first to report cases of KD in Saudi Arabia, with three cases were saudi nationals.

From the capital, that is, the central region, Ghazal *et al*. [3] studied 29 patients with KD at Sulaimania Children’s Hospital in Riyadh for 8 years. Muzaffer *et al*. reviewed 13 medical records/referral letters of patients diagnosed with KD at King Faisal Specialist Hospital and Research Center, Riyadh (1997–2001). None of them reported having AKI [7].

Alsaggaf *et al*. analyzed 56 children diagnosed with atypical KD for over 12 years [17]. Khalid Alharbi studied 51 patients suspected of having KD [5], and Lardhi also studied 35 patients [6], none of whom had AKI. These studies took place in the western and eastern parts of the Kingdom.

In addition, a retrospective study was conducted in the southwest of the Kingdom (Albaha), which included 40 children with KD, none of whom reported having AKI [21].

On the contrary, AKI is not uncommon in the Kingdom. In a recent multicenter prospective cohort study by the Kingdom of Saudi Arabia (KSA) conducted using the KDIGO definition, 37.4% of critically ill children had AKI. Although sepsis, infections, and postcardiac surgery were the most common causes, KD was not reported as an etiology [10].

AKI is usually divided into three broad groups depending on the cause: prerenal AKI, described by reduced kidney perfusion in cases with no parenchymal injury; renal parenchymal injuries causing renal AKI; and postrenal AKI caused by obstruction of the urinary tract [10].

Both prerenal and renal AKI have been reported in patients with KD. Tubular interstitial nephritis, hemolytic uremic syndrome, immune-complex mediated nephropathy, and KD shock syndrome have been described as causes of renal AKI. Meanwhile, acute congestive heart failure and fluid loss have been described as causes of prerenal AKI [8, 10, 14, 15].

AKI in our patient may have been due to prerenal AKI, which is secondary to dehydration, as there was evidence of poor intake for several days before presentation, which explains the cracked lips and poor appetite. The kidney biopsy was not performed when the patient’s kidneys opened, and the renal function normalized with the beginning of AKI management.

Treatment of KD patients with prerenal AKI involves the appropriate restoration of normal circulating blood volume, and it must be adjusted to the severity of AKI through fluid restriction, diuretic use, and renal replacement therapy, in addition to the specific therapy for KD [10]. This describes the response of the intravenous bolus and Lasix, intravenous immunoglobulin, and aspirin [22]. Fortunately, the recovery was achieved without dialysis.

Kari [23] showed that AKI is associated with increased mortality after discharge, and oliguria is a predictor that increases mortality [10].

Regular follow-up appointments were given to the patient, and the parents were informed of its importance.

## Conclusions

In summary, a lack of reporting and/or routine renal function tests could make AKI unusual in KD. Checking the SCr level is worthwhile when KD is suspected, or to confirm a case of KD to reduce the harmful consequences of late discovery. Early detection and intervention make a substantial difference in AKI management. Further, larger multicenter studies are needed to determine whether AKI in KD varies by geographical area and between age groups. This will facilitate the identification of the association and help avoid unnecessary diagnostic tests.

## Data Availability

Not applicable

## Abbreviations

AKI: Acute kidney injury
COVID-19: Coronavirus disease
GFR: Glomerular filtration rate
KD: Kawasaki disease
MIS-C: Multisystemic inflammatory syndrome in children
SARS-CoV-2: Severe acute respiratory syndrome coronavirus 2

## References

[1] Saviour MJ, Hassan S (2017). Kawasaki disease presenting with bloody diarrhea and acute renal failure: first case. Pediatr Rep.

[2] Mousa FM, Michail EA, El-Sowailem AM (1989). Kawasaki syndrome in Saudi children. Ann Saudi Med.

[3] Ghazal SS, Alhowasi M, El Samady MM (1998). Kawasaki disease in a pediatric hospital in Riyadh. Ann Trop Pediatr.

[4] Yang HF, Chen WL, Chang CN, Chen SJ, Fan HC (2016). Kawasaki disease shock syndrome: case report. Pediatr Int Child Health.

[5] Al-Harbi KM (2010). Kawasaki disease in Western Saudi Arabia. Saudi Med J.

[6] Lardhi AA (2013). Kawasaki disease: a university hospital experience. Saudi J Med Sci.

[7] Muzaffer MA, Al-Mayouf SM (2002). Pattern of clinical features of Kawasaki disease. Saudi Med J.

[8] Watanabe T (2013). Kidney and urinary tract involvement in Kawasaki disease. Int J Pediatr.

[9] Lazea C, Man O, Sur LM, Serban R, Lazar C (2019). Unusual presentation of Kawasaki disease with gastrointestinal and renal manifestations. Ther Clin Risk Manag.

[10] Watanabe T (2018). Clinical features of acute kidney injury in patients with Kawasaki disease. World J Clin Pediatr.

[11] Mac Ardle BM, Chambers TL, Weller SDV, Tribe CR (1983). Acute kidney injury in Kawasaki disease. J R Soc Med.

[12] Chuang GT, Tsai IJ, Lin MT, Chang LY (2016). Acute kidney injury in patients with Kawasaki disease. Pediatr Res.

[13] Khwaja A (2012). KDIGO clinical practice guidelines for acute kidney injury. Nephron Clin Pract.

[14] Al-Saeed G, Rizk T (2015). Refractory Kawasaki disease: unusual presentation and mini review. J Pediatr Neonatal Care.

[15] Tiewsoh K, Sharma D, Jindal AK, Bhisikar S, Suri D, Singh S (2018). Acute kidney injury in Kawasaki disease, report of 3 cases from north India and a brief review of literature. J Clin Rheumatol.

[16] Nugud AA, Nugud A, Wafadari D, Abuhammour W (2019). Kawasaki shock syndrome in an Arab female: case report of a rare manifestation and review of literature. BMC Pediatr.

[17] Alsaggaf HM (2013). Clinical experience of Kawasaki disease in two tertiary care centers in Jeddah, Saudi Arabia. Med Sci.

[18] Sleiman R, Almohayya T, Al Hennawi H (2019). Unusual presentation of Kawasaki disease in a 13-year-old Saudi boy. Cureus.

[19] Multisystemic inflammatory syndrome in children (MIS-C) CDC guidelines.

[20] Lang B (2001). Recognizing Kawasaki disease. Pediatr Child Health.

[21] Almawazini AM, Alnashi S, Alsharkawy AA, Almawazini MA, Almawazini HA, Alzahrani MS, Alqahtani SAM (2019). Overview of Kawasaki disease in Albaha area, Saudi Arabia. JHMS.

[22] Barut K (2016). Pediatric vasculitis. Curr Open Rheumatol.

[23] Kari JA. Epidemiology of acute kidney injury in critically ill children living in the Kingdom of Saudi Arabia. Asian J Pediatr Nephrol. 2018;1:52–5.

